# Detection of Novel Human Astrovirus MLB1 by a Commercial Viral Gastroenteritis Multiplex Assay

**DOI:** 10.1128/spectrum.00469-22

**Published:** 2022-05-23

**Authors:** Yolanda I. I. Ho, Christine W. S. Lee, Ann H. Wong

**Affiliations:** a Department of Microbiology, Prince of Wales Hospital, Hong Kong, China; University of California, San Diego

**Keywords:** classic human astrovirus, commercial multiplex assay, novel human astrovirus

## LETTER

Classic human astrovirus (HAstV-C) types 1 to 8 have been shown to be causative agents of viral gastroenteritis, mainly affecting children, elderly patients, and immunocompromised patients (1). HAstV-MLB types 1 to 3 and the closely related HAstV-VA/HMO types 1 to 5 are often referred to as novel HAstV types and have been identified in fecal samples from patients with gastroenteritis (2). Novel HAstV types are phylogenetically more closely related to animal astroviruses than to HAstV-C, suggesting cross-species transmission (3). Novel HAstV types, including MLB1, MLB2, and VA1/HMO-C, were originally described in association with severe neurological infections and have been reported to be the causative agents of encephalitis/encephalopathy and meningitis in immunocompromised patients (4).

Between August 2020 and August 2021, we conducted an evaluation of the BD Max enteric viral panel (EVP). A total of 5,055 stool samples from hospitalized patients with gastroenteritis were tested. This commercial multiplex PCR assay qualitatively detects and differentiates enteric viral pathogens, including norovirus GI and GII, rotavirus A, HAstV, adenovirus F40/41, and sapovirus genogroups I, II, IV, and V. Fourteen tested samples from 13 patients were HAstV positive. These HAstV-positive samples were retrieved from storage at −70°C and were retrospectively tested with a monoplex real-time assay (VEB-HAst-C) for HAstV-C and with a multiplex real-time assay (VEB-HAst-MLB and VEB-HAst-VA/HMO) for novel HAstV types, as designed by Nijhuis et al. (5). The methodology was the same as the one reported by Nijhuis et al. except that RNA was extracted with the MagMax 96 viral RNA kit (Ambion, USA) and real-time PCR was performed with the ABI 7900 fast sequence detection system (Thermo Fisher Scientific, USA). Five of the samples were HAstV-C positive, and 4 were HAstV-MLB positive. The remaining 5 samples were negative by both assays, one is a monoplex real-time assay for HAst-C and the other one is a multiplex real-time assay for novel HAst (Table 1).

**TABLE 1:** Patient demographic characteristics and real-time PCR results for the 13 BD Max EVP-HAstV-positive cases

Patient	Sex^*a*^	Age	HAstV detection with:			
			BD Max EVP	VEB-HAst-C PCR	VEB-HAst-MLB multiplex PCR	VEB-HAst-VA/HMO multiplex PCR
1	F	26 yr	Detected (*C_T_* = 15)	Not detected	Detected (*C_T_* = 13.73)	Not detected
2	F	65 yr	Detected (*C_T_* = 20)	Not detected	Detected (*C_T_* = 15.40)	Not detected
	F	65 yr	Detected (*C_T_* = 27)	Not detected	Detected (*C_T_* = 28.72)	Not detected
3	M	18 yr	Detected (*C_T_* = 28)	Not detected	Detected (*C_T_* = 29.09)	Not detected
4	F	11 mo	Detected (*C_T_* = 25)	Detected (*C_T_* = 17.34)	Not detected	Not detected
5	F	8 mo	Detected (*C_T_* = 19)	Detected (*C_T_* =14.75)	Not detected	Not detected
6	F	16 yr	Detected (*C_T_* = 18)	Detected (*C_T_* = 6.62)	Not detected	Not detected
7	F	1 yr	Detected (*C_T_* = 15)	Detected (*C_T_* =12.14)	Not detected	Not detected
8	F	5 yr	Detected (*C_T_* = 16)	Detected (*C_T_* = 8.60)	Not detected	Not detected
9	F	67 yr	Detected (*C_T_* = 34)	Not detected	Not detected	Not detected
10	M	81 yr	Detected (*C_T_* = 31)	Not detected	Not detected	Not detected
11	M	3 yr	Detected (*C_T_* = 36)	Not detected	Not detected	Not detected
12	F	75 yr	Detected (*C_T_* = 36)	Not detected	Not detected	Not detected
13	F	88 yr	Detected (*C_T_* = 35)	Not detected	Not detected	Not detected

a F, female; M, male.

To differentiate the HAstV-MLB types identified in the three adult patients (patients 1 to 3), primers SF0073 and SF0076, tagged with M13-F and M13-R, respectively, were used to amplify a 409-bp fragment of the open reading frame 1b (ORF1b) gene (RNA-dependent RNA polymerase) from the corresponding RNA with the use of one-step reverse transcription (RT)-PCR (2). The PCR product from each patient was then sequenced with M13-F and M13-R in both directions using the ABI Prism 3500 genetic analyzer and BigDye Terminator cycle sequencing kit v3.1 (Applied Biosystems, Thermo Fisher Scientific).

**FIG 1:**
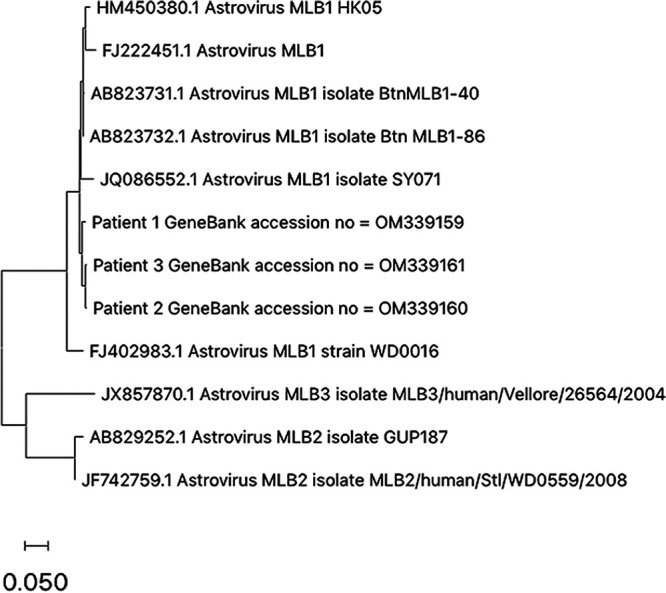
Phylogenetic analysis based on alignment of a region of the ORF1b sequences (409 bp) of HAstV-MLB types 1 to 3. The tree was generated by the maximum likelihood method with 1,000 replicates. Both reference sequences and the sequences for the patients are identified with their GenBank accession numbers.

Nine reference sequences belonging to MLB1, MLB2, and MLB3 were downloaded from GenBank and were aligned with the three consensus sequences with MEGA X v11. A phylogenetic tree using the maximum likelihood method and 1,000 bootstrap replicates was also constructed with MEGA X. The three cases of HAstV-MLB were confirmed as MLB1 (Fig. 1).

Primer sequences of the BD Max EVP are not disclosed. The manufacturer states that only HAstV-C types 1 to 8 and an unknown strain are included in its analytical inclusivity. However, we confirmed that this panel can detect HAstV-MLB1. Whether other novel HAstV types can be detected remains uncertain. Patients 9 to 13, who tested negative by the two real-time PCRs, also tested negative by HAstV-C one-step RT-PCR and a novel HAstV one-step RT-PCR designed by Finkbeiner et al. (2). Review with the BD Max software showed that their amplification occurred at very high threshold cycle (*C_T_*) values (Table 1). Repeat testing of frozen archives of these 5 samples with the EVP yielded negative results. The relatively low viral load in these samples and RNA degradation due to prolonged sample storage with freezing and thawing may account for such nonreproducible EVP results, as well as their discrepancies with the real-time PCR results.

In conclusion, HAstV-MLB1 is circulating in Hong Kong and can be detected with BD Max EVP. To better understand its epidemiology and pathogenic role, further studies are warranted.
